# Supplementary material for: Epithelial plasticity and innate immune activation promote lung tissue remodeling following respiratory viral infection
Source: Nat Commun. 2023 Sep 19;14:5814. doi: 10.1038/s41467-023-41387-3 (PMC10509177; doi:10.1038/s41467-023-41387-3)
Supplement: Supplementary file 1 — Supplementary Information [file 41467_2023_41387_MOESM1_ESM.pdf]

Supplementary Table 1.

REAGENT or RESOURCE	SOURCE	IDENTIFIER
Antibodies		
Primary antibodies for IF:		
Chicken polyclonal anti-eGFP (1:1000)	Abcam	Ab13970
Chicken Polyclonal anti-Keratin 5 (1:500)	BioLegend	905901
Mouse monoclonal anti-eGFP AF488 (1:500)	Santa Cruz	Sc-9996
Rat monoclonal anti-IL-22ra1(1:200)	R&D systems	MAB42341
Goat polyclonal anti-tdTomato (1:500)	Scigen	Ab8181-200
Goat polyclonal anti-p63 (1:500)	Santa Cruz	Sc-8609
Goat Polyclonal Ugrp1/Scgb3a2 (1:1000)	R&D Systems	AF3465
Syrian hamster Monoclonal anti-Pdpn (1:1000)	LifeSpan Biosciences	LS-C143022-100
Rabbit Polyclonal anti-Scgb1a1 (1:500)	Proteintech	10490-1-AP
Rabbit Polyclonal anti-tdT (1:500)	Rockland	600-401-379
Rabbit Polyclonal anti-Msln (1:500)	Thermo Fisher Scientific	PA5-79698
Rabbit Polyclonal anti-Ltf (1:200)	Thermo Fisher Scientific	PA5-95513
Rabbit Polyclonal anti-Bpifa1 (1:200)	Sigma-Aldrich	AV42475
Rabbit polyclonal anti-Keratin 5 (1:500)	Cell Marque	EP1601Y
Rabbit polyclonal anti-Keratin 5 (1:500)	Santa Cruz	Sc-66856
Rabbit polyclonal anti IL-22 (1:200)	Abcam	ab18499
Rabbit polyclonal anti-Ki67 (1:1000)	ebioscience	14-5698-82
Secondary antibodies for IF:		
Goat anti-Chicken Alexa Fluor 488 (1:500)	Thermo Fisher Scientific	6100-30
Goat anti-Hamster Alexa Fluor 488 (1:500)	Thermo Fisher Scientific	A-21110
Donkey anti-Rabbit Alexa Fluor 488 (1:500)	Thermo Fisher Scientific	A-21206
Donkey anti-Goat Alexa Fluor 555 (1:500)	Thermo Fisher Scientific	A-21432
Donkey anti-Rabbit Alexa Fluor 555 (1:500)	Thermo Fisher Scientific	A-31572

Goat anti-Chicken Alexa Fluor 568 (1:500)	Thermo Fisher Scientific	A-11041
Donkey anti-Rat Alexa Fluor 594 (1:500)	Thermo Fisher Scientific	A-21209
Donkey anti-Goat Alexa Fluor 594 (1:500)	Thermo Fisher Scientific	A-11058
Donkey anti-Rabbit Alexa Fluor 594 (1:500)	Thermo Fisher Scientific	A-21207
Goat anti-Hamster Alexa Fluor 594 (1:500)	Thermo Fisher Scientific	A-21113
Goat anti-Chicken Alexa Fluor 647 (1:500)	Thermo Fisher Scientific	A-21449
Donkey anti-Rabbit Alexa Fluor 647 (1:500)	Thermo Fisher Scientific	A-31573
Donkey anti-Goat Alexa Fluor 647 (1:500)	Thermo Fisher Scientific	A-21447
Primary antibodies for Flow:		
Fitc rat monoclonal anti-CD45 (1:200)	BioLegend	103108
Fitc rat monoclonal anti-CD31 (1:200)	BioLegend	102406
APC rat monoclonal anti-CD326 (1:200)	BioLegend	118218
Pe/Cy7 rat monoclonal anti-CD326 (1:200)	BioLegend	118216
Biotin monoclonal anti-CD31 (1:200)	BioLegend	102404
Biotin monoclonal anti-CD45 (1:200)	BioLegend	103104
Biotin monoclonal anti-Ly-6A/E (1:200)	BioLegend	108112
TotalSeq Hashtag 1 Antibody (1:200)	BioLegend	155801
TotalSeq Hashtag 2 Antibody (1:200)	BioLegend	155803
TotalSeq Hashtag 3 Antibody (1:200)	BioLegend	155805
TotalSeq Hashtag 4 Antibody (1:200)	BioLegend	155807
Bacterial and virus strains		
Influenza A virus (H1N1)	ATCC	VR-95
Chemicals, peptides, and recombinant proteins		
Antigen Unmasking Solution, Tris-Based	Vector Laboratories	H-3301
Antigen Unmasking Solution, Citric Based	Vector Laboratories	H-3300
Tamoxifen, ≥99%	Sigma-Aldrich	T5648-5G
Elastase	Worthington Biochemical	LS002280
Liberase™ TM Research Grade	Sigma-Aldrich	5401127001
1X RBC Lysis Buffer	eBioscience	00-4333-57
Critical commercial assays		
Lysing matrix D 2 mL tubes	MP Biomedicals	116913100
Bio-plex 200	Luminex	171000201
Bio-plex Pro 6-plex	Bio-Rad	171304070
Bio-plex Pro 1-plex (custom)	Bio-Rad	M69999997NY

MP Benchtop Homogenizer	MP Biomedicals	6VFV9
RNAeasy mini kit	Qiagen	74106
IScript cDNA synthesis kit	Bio-Rad	1708891
SYBR Green PCR master Mix	Thermo Fisher Scientific	4309155
Deposited data		
Raw and analyzed data	This manuscript	GSE184384
Experimental models: Organisms/strains		
B6.129S-Sftpctm1(cre/ERT2)Blh/J	Source: Jackson Labs; (Rock et al., 2011)	028054
B6N.129S6(Cg)-Scgb1a1tm1(cre/ERT)Blh/J	Source: Jackson Labs; (Rawlins et al., 2009)	016225
B6N.129S6(Cg)-Krt5tm1.1(cre/ERT2)Blh/J	Source: Jackson Labs; (Van Keymeulen et al., 2011)	029155
C57BL/6-Il22tm1.1(icre)Stck/J	Source: Jackson Labs; (Ahlfors et al., 2014)	027524
B6.Cg-Il22ra1tm1.1Koll/J	Source: Jackson Labs; (Zheng et al., 2016)	031003
B6.Cg-Shhtm1(EGFP/cre)Cjt/J	Source: Jackson Labs; (Harfe et al., 2004) 027524	005622
B6.129(Cg)-Gt(ROSA)26Sortm4(ACTB-tdTomato,-EGFP)Luo/J	Source: Jackson Labs; (Muzumdar et al., 2007)	007576
B6.Cg-Gt(ROSA)26Sortm9(CAG-tdTomato)Hze/J	Source: collaborator; (Madisen et al., 2010)	007905
B6.Cg-Gt(ROSA)26Sortm1.2(CAG-tdTomato,-EGFP)Pjen/J	Source: Jackson Labs;(Plummer et al., 2015)	026931
Scgb3a2-DreER (generated using a BL6/j genetic background)	Source: Jackson Labs	NA
Software and algorithms		
Fiji image analysis software	(Schindelin et al., 2012)	https://imagej.net/software/fiji/
Graphpad Prism7	GraphPad	https://www.graphpad.com/scientific-software/prism/
Illustrator	Adobe	https://www.adobe.com/products/illustrator.html
Zen2	Zeiss	https://www.zeiss.com/microscopy/us/products/microscope-software/zen.html

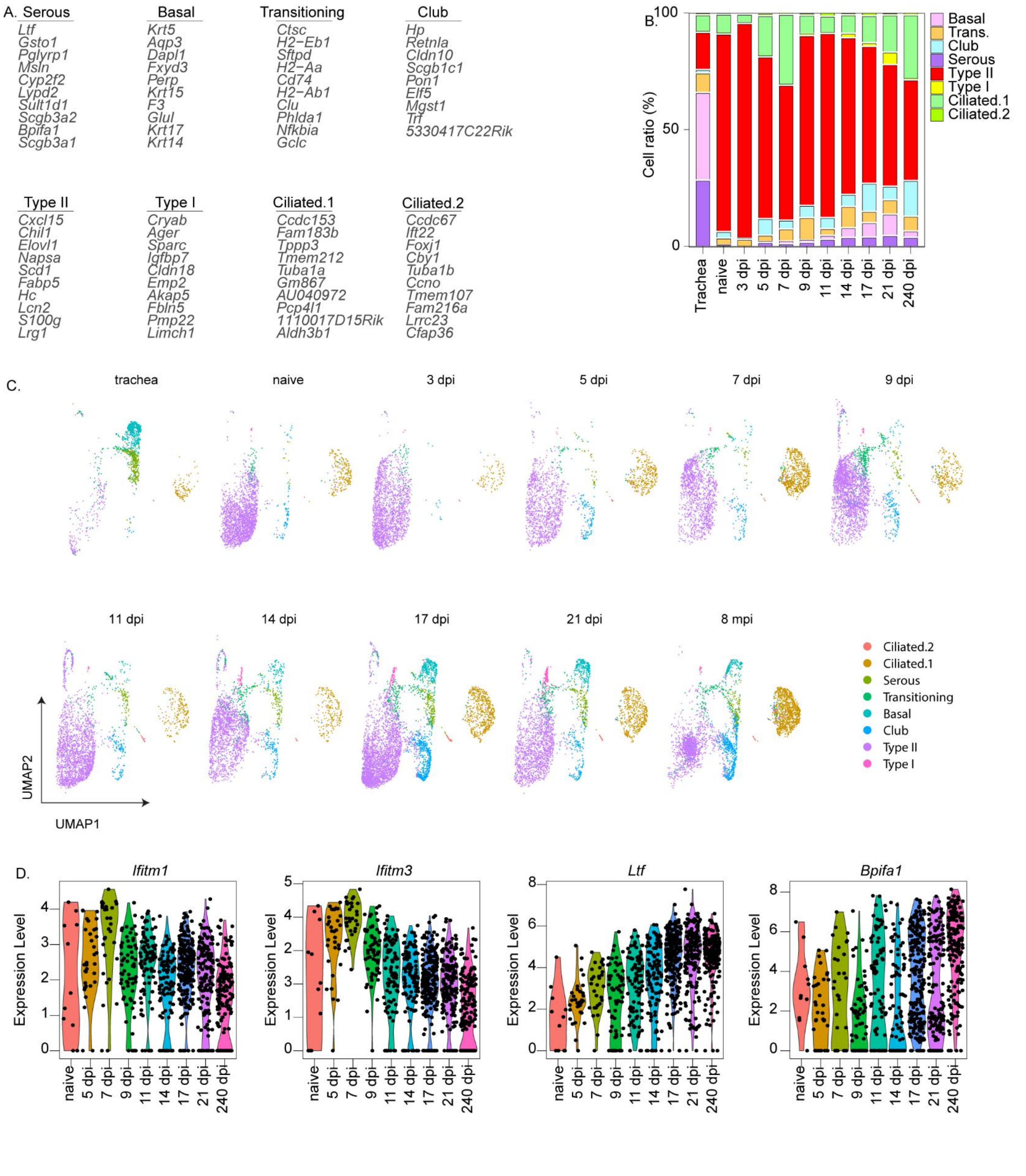

Supp. figure 1

Supplementary Fig. 1. Club and IS cell cluster independently of cell cycle genes.

(A) Full gene list for heatmap shown in Fig 1. A.
(B). Representation of lung epithelial cell types as a function of percent total sampled cells at each time point. dpi = days post infection.
(C) UMAP stratified by timepoints post-PR8 infection.
(D) Violin plot comparing expression of selected antimicrobial genes at indicated time points after PR8 infection.

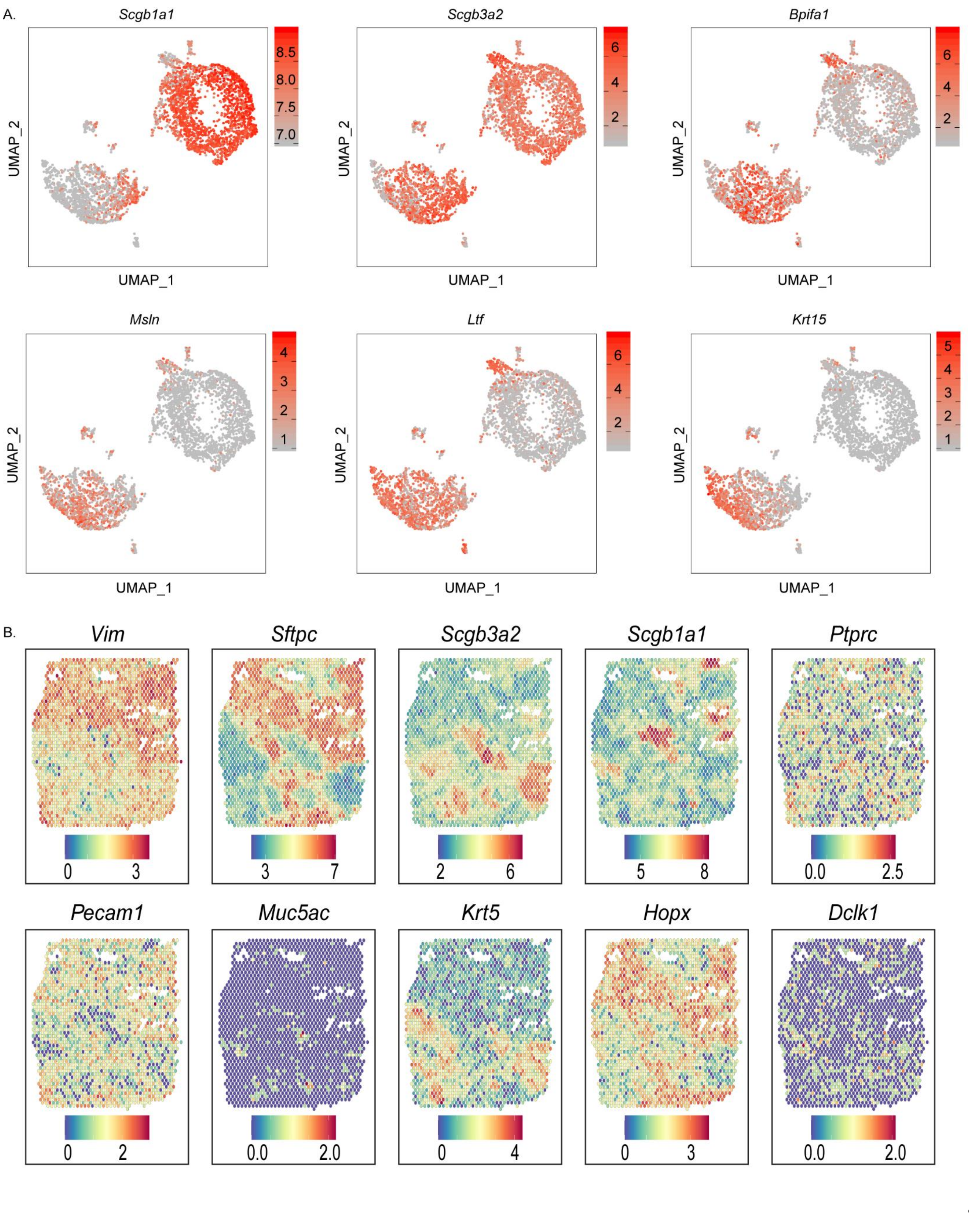

Supp. figure 2

Supplementary Fig. 2. Assessment of transcriptional differences between club and IS populations.

(A) Feature plot comparing expression of selected club and serous cell-specific genes between cell types.
(B) Spatial feature plot comparing expression of selected cell-specific genes at 14 days following PR8 exposure: *Krt5* (basal), *Scgb3a2* (serous and club), *Bpifa1* (serous), *Muc5ac* (goblet), *Scgb1a1* (club), *Vim* (Fibroblast), *Pecam1* (endothelial), *Ptprc* (Immune).

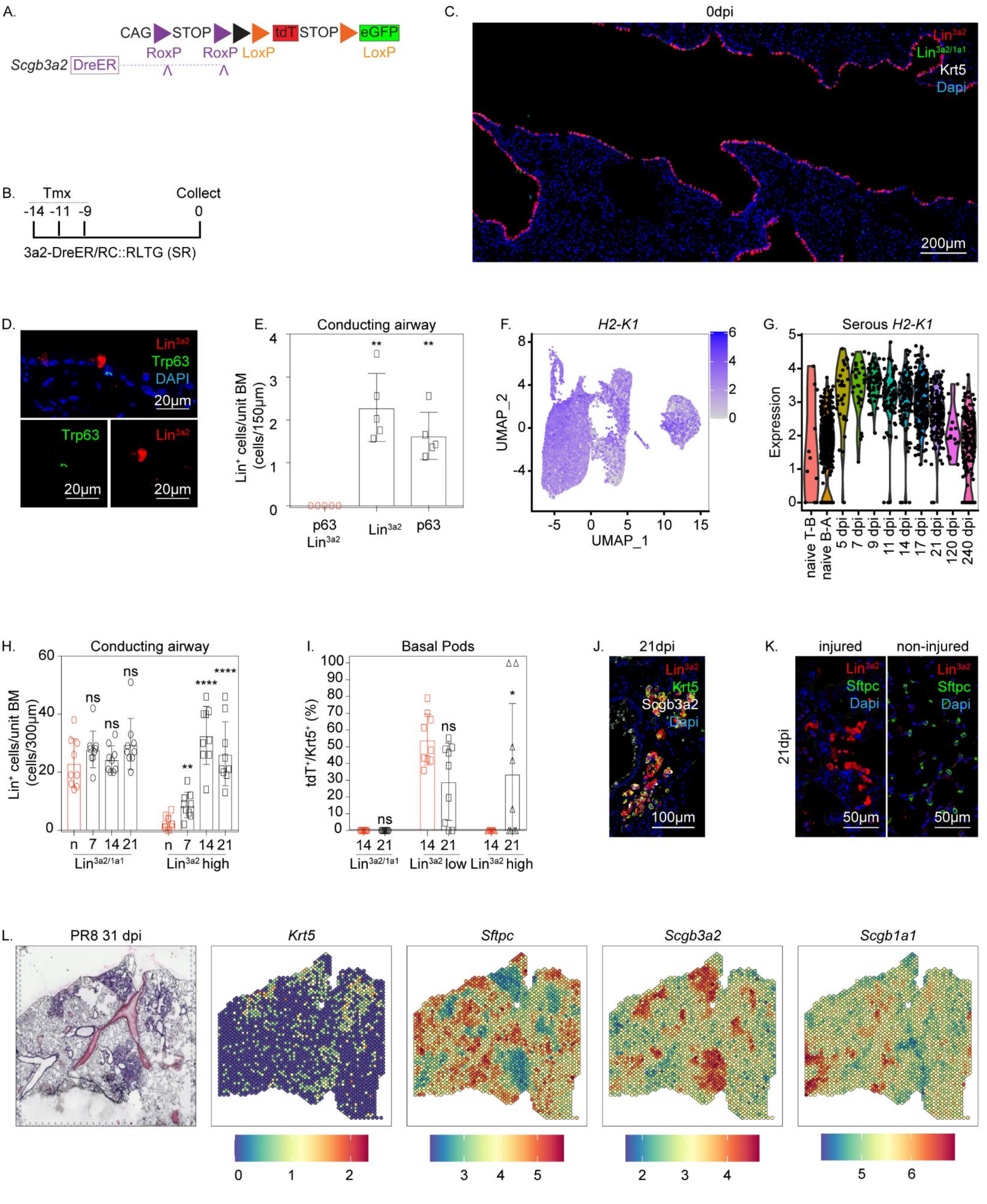

Supp. figure 3

Supplementary Fig. 3. Further characterization of DR mice in the context of influenza induced lung injury

(A) Schematic illustration of recombinase driver and reporter allele used in Dre recombinase (SR) mice.
(B) Experimental design. SR mice (n = 5 per experimental group) were treated with 3 doses of TM to assess degree of non-specific recombination.
(C) Representative immunofluorescence localization of lineage reporters (green or red) with Krt5 (white) in SR mice at steady state.
(D) Representative immunofluorescence colocalization of tdT (Lin^{3a2}; red) with Trp63 (green) within conducting airway epithelium at steady state.
(E) Contribution of lineage-tagged populations to Trp63+ progenitors within conducting airway epithelium at steady state. Data are presented as mean values +/- SEM. n=3 biologically independent samples per condition, with significance determined by Mann-Whitney two-tailed U-test (** P < 0.01). Source data are provided as a Source Data file.
(F) Feature plot showing H2-K1 expression between cell types.
(G) Violin Plot comparing expression of H2-K1 within subsetted serous cells at indicated time points after PR8 infection.
(H) Contribution of lineage-tagged populations to hBC in airways as a function of time after PR8 infection . Data are presented as mean values +/- SEM. n=3 biologically independent samples per condition, with significance determined by Mann-Whitney two-tailed U-test (** P < 0.01, **** P < 0.0001). Source data are provided as a Source Data file.
(I) Contribution of lineage-tagged populations to hBC in alveolar ‘pods’ as a function of time after PR8 infection . Data are presented as mean values +/- SEM. n=3 biologically independent samples per condition per condition, with significance determined by Mann-Whitney two-tailed U-test (* P < 0.05). Source data are provided as a Source Data file.
(J) Representative immunofluorescence colocalization of tdT (Lin^{3a2}; red) with Krt5 (green) & Scgb3a2 (white) among lineage-positive alveolar clusters 21 days after PR8 infection.
(K) Representative immunofluorescence colocalization of tdT (Lin^{3a2}; red) with Sftpc (green) among lineage-positive alveolar clusters 21 days after PR8 infection.
(L) Spatial gene expression and corresponding immunofluorescence of cell type-specific markers 31 days following exposure to PR8: *Krt5* (basal), *Scgb3a2* (serous and club), *Scgb1a1* (club) and *Sftpc* (AT2).

A.

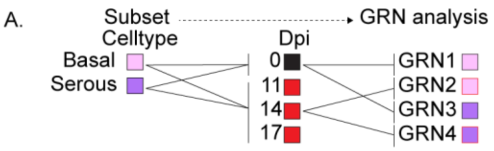

B.

	Nodes	Edges
GRN1: Naive_Basal	3886	7632
GRN2: Late_Basal	3233	7800
GRN3: Naive_Serous	5017	14624
GRN4: Late_Basal	4642	14505

C.

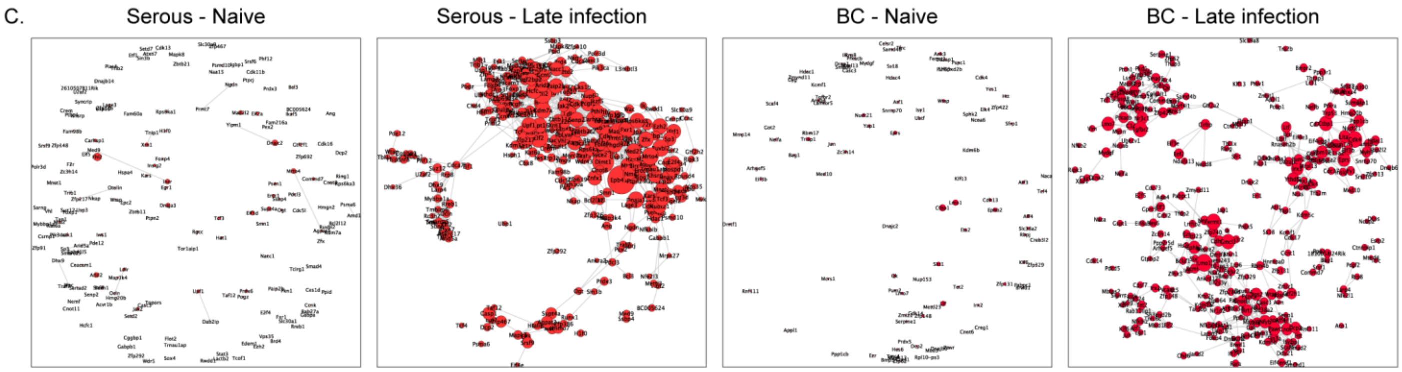

D.

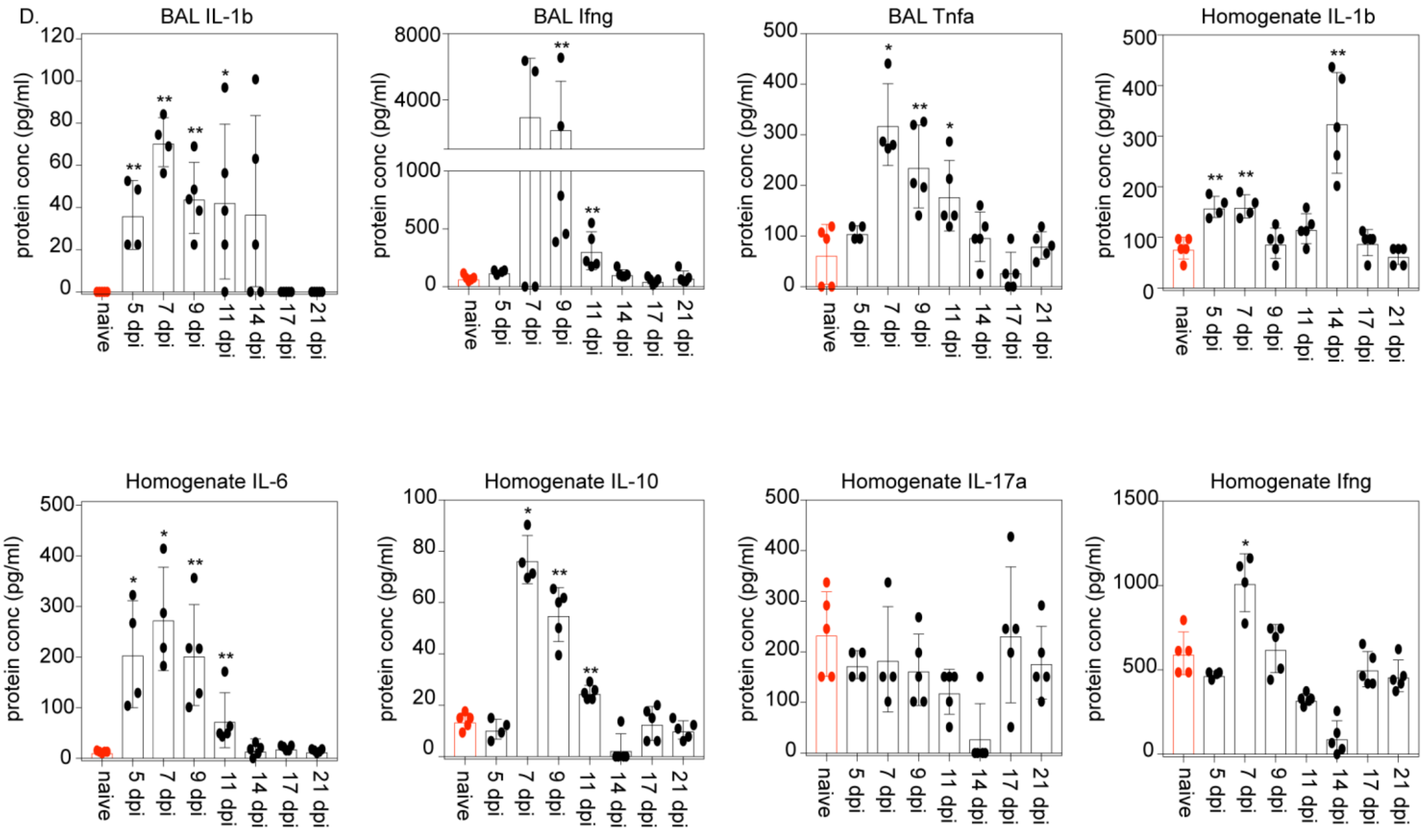

Supplementary Fig. 4 Gene regulatory network analysis of PR8 infected BC reveal increased immunoreactivity following injury.

(A) Experimental design for generation of GRNs. ScRNAseq data were first subsetted by cell type followed by segregation into early inflammatory phase, peak inflammation, and recovery , resulting in the creation of four networks for downstream analysis. 392 Serous and 513 BC were input for early timepoints; 412 Serous and 462 BC were input for late timepoints.
(B) Table showing node and edge number after homogenization of late time points to their respective naïve controls.
(C) Visualization of GRNs. Node and font sizes reflect degree centrality and strength of connectivity. The top five genes with the highest delta centrality value relative to naïve controls are highlighted in green, with remaining genes shown in red.
(D) Assessment of cytokine levels in BALF & lung homogenate from PR8 infection. Data are presented as mean values +/- SEM. n = 4 for 5 and 7 day post-exposure conditions. n = 5 for all other conditions. All analyzed samples were biologically independent. were used per timepoint with statistical significance determined by Mann-Whitney two-tailed U-test (* $P < 0.05$, ** $P < 0.01$). Source data are provided as a Source Data file.

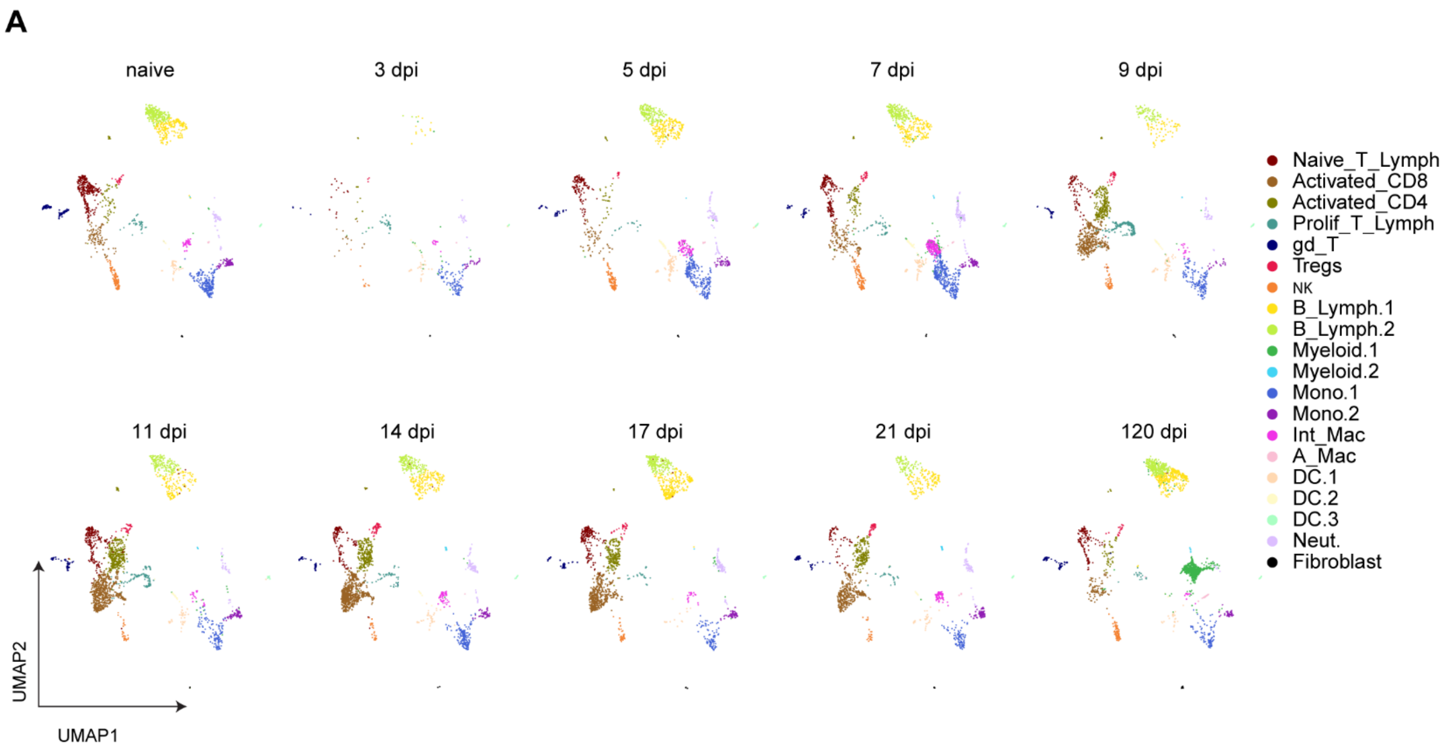

Supp. figure 5

Supplementary Fig. 5. Changes in immune cell populations following PR8 infection.
(A) UMAP stratified by timepoints post-PR8 infection.

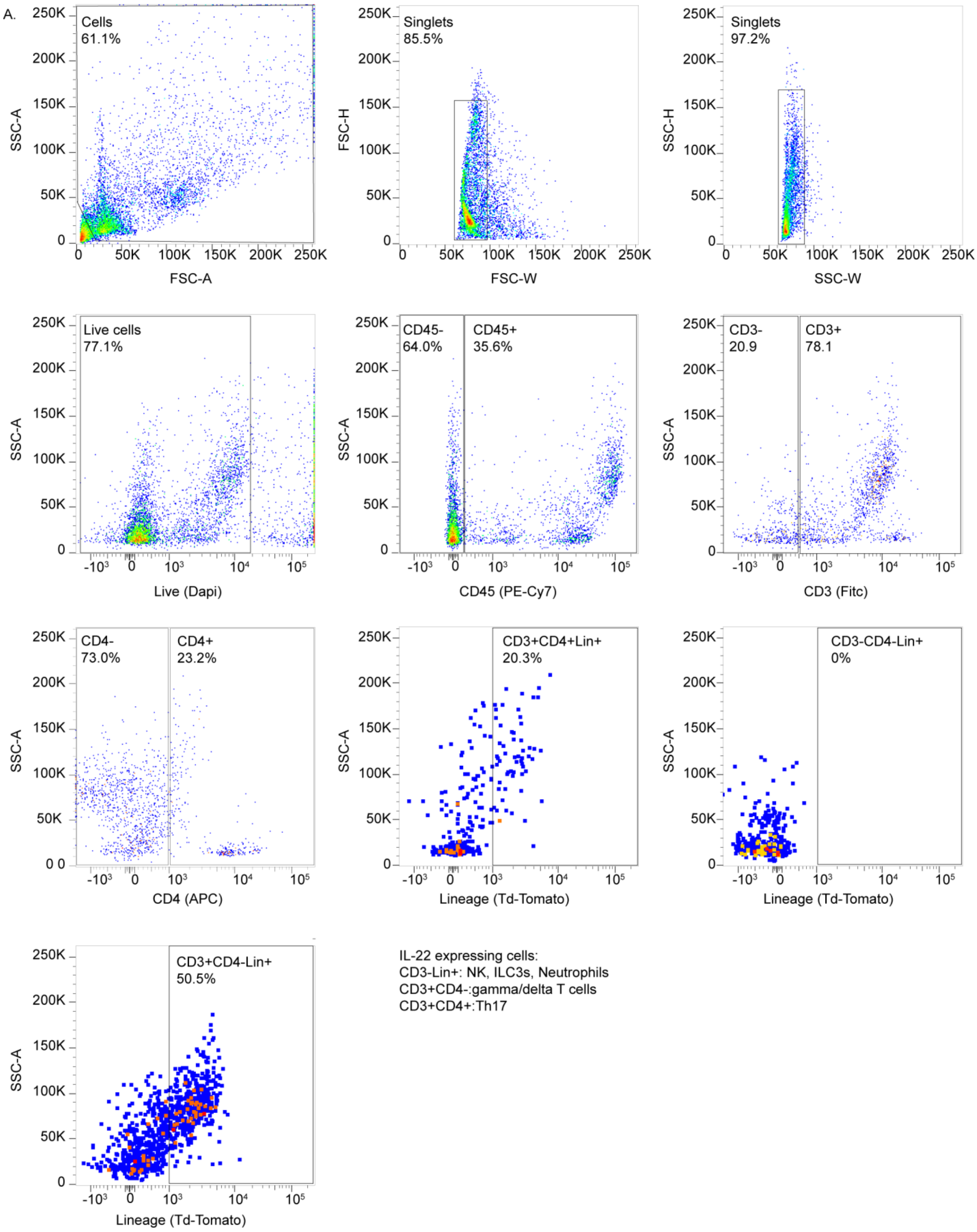

IL-22 expressing cells:
CD3-Lin+: NK, ILC3s, Neutrophils
CD3+CD4-:gamma/delta T cells
CD3+CD4+:Th17

Supplementary Fig. 6. Gating strategy for quantification of IL-22 expressing subsets using flow cytometry.

(A) Gating strategy to assess different IL-22 expressing cell types via flow cytometry. Cells were subsetted into $CD3^{-}Lin^{+}$, $CD3^{+}CD4^{-}Lin^{+}$ and $CD3^{+}CD4^{+}Lin^{+}$ populations to delineate non-T lymphoid, γδT and Th17 cells respectively.

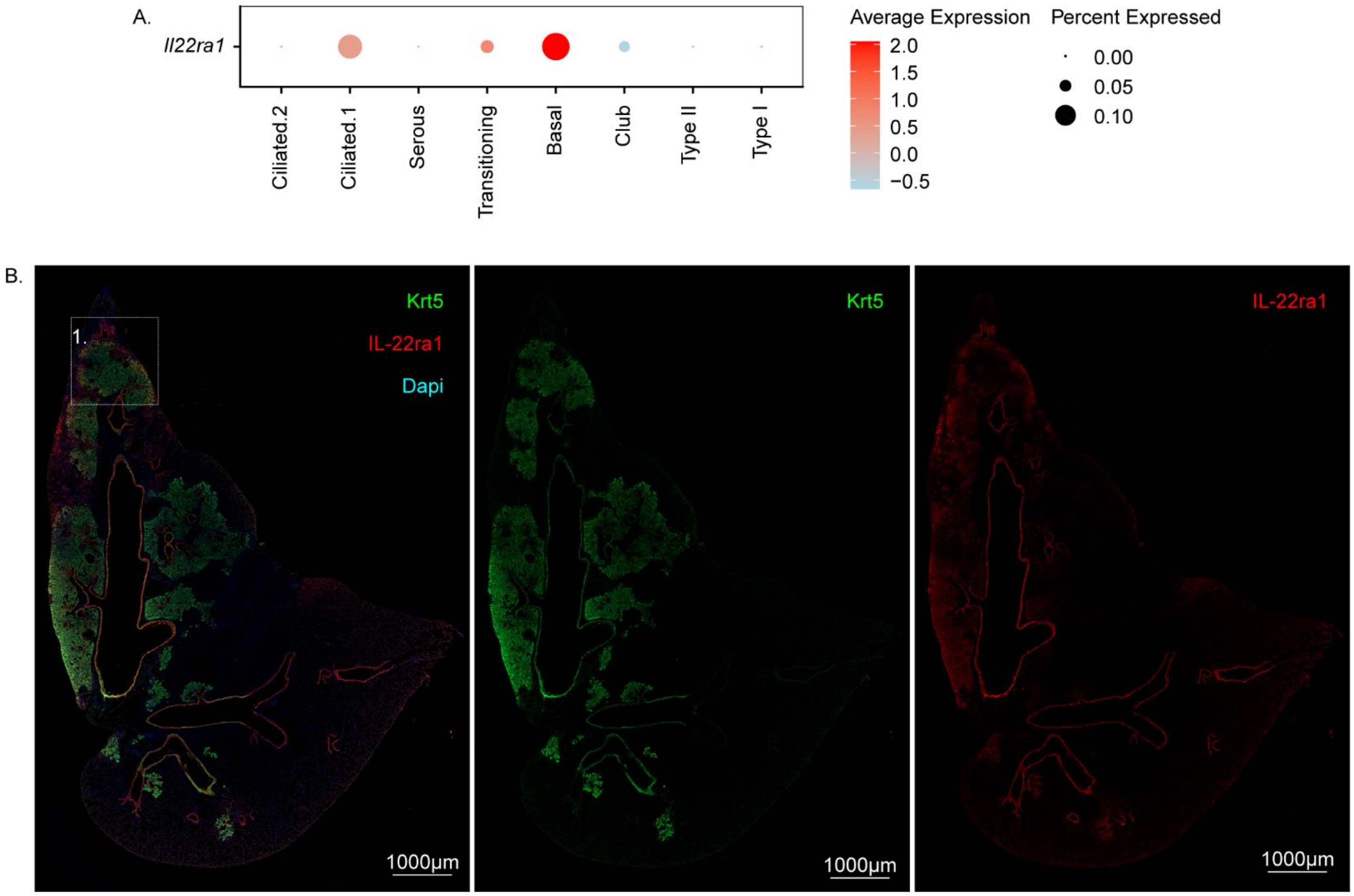

Supp. figure 7

Supplementary Fig. 7. Visualization of IL-22ra1 positive and negative BC regions during repair.

(A) Dot plot comparing expression of IL-22ra1 between epithelial subsets.
(B) Representative immunofluorescent colocalization of IL-22ra1 (red) and Krt5 (green) in BC-rich alveolar region of PR8-infected mouse lung.

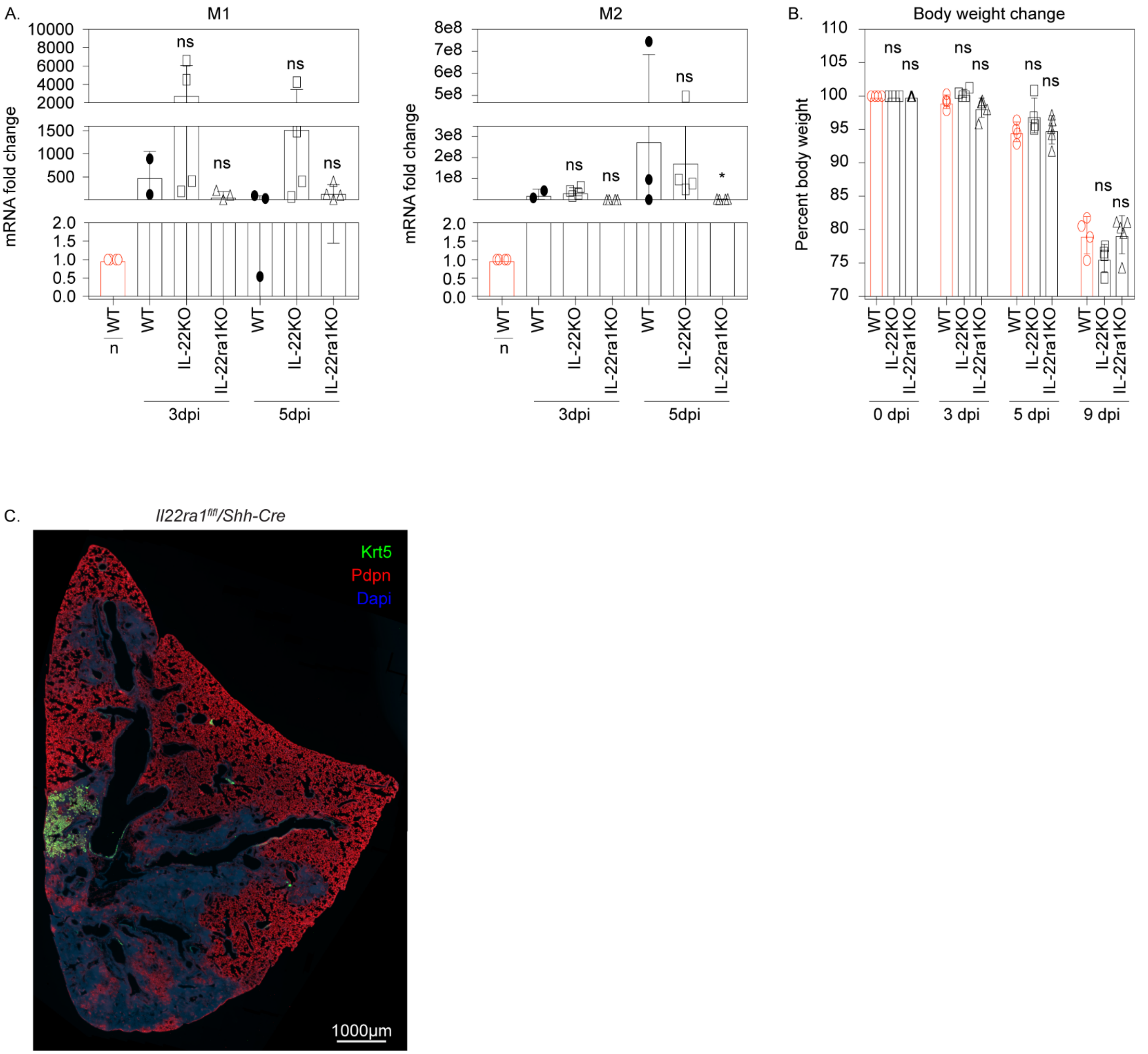

Supp. figure 8

Supplementary Fig. 8. Additional phenotyping for IL-22 LOF & IL-22r cLOF

(A) qPCR detection of viral gene mRNA in total lung RNA of PR8-infected WT, IL-22 LOF, and IL-22r cLOF mice. Data are presented as mean values +/- SEM. n = 2, 4, 4, 3, 4, 4 for 3dpi WT/IL-22 LOF/IL-22r cLOF and 5dpi WT/IL-22 LOF/IL-22r cLOF conditions respectively. All analyzed samples were biologically independent. Statistical analysis was performed by Mann-Whitney two-tailedU-test.
(B) Body weight changes of WT, IL-22 LOF, and IL-22r cLOF mice at different points during influenza induced acute lung injury. Data are presented as mean values +/- SEM. n=5 for IL-22r cLOF condition. n=4 for WT and IL-22 LOF conditions. All analyzed samples were biologically independent. Statistical analysis was performed by Mann-Whitney two-tailed U-test.
(C) Representative immunofluorescence localization of Pdpn (red) and Krt5 (green) in lungs of IL-22r cLOF mice 14 days post-PR8 infection.
