# Supplementary material for: Epithelial plasticity and innate immune activation promote lung tissue remodeling following respiratory viral infection
Source: Nat Commun. 2023 Sep 19;14:5814. doi: 10.1038/s41467-023-41387-3 (PMC10509177; doi:10.1038/s41467-023-41387-3)
Supplement: Supplementary file 3 — Reporting Summary [file 41467_2023_41387_MOESM3_ESM.pdf]

nature portfolio

Corresponding author(s): Barry R. Stripp

Last updated by author(s): Aug 5, 2023

Reporting Summary

Nature Portfolio wishes to improve the reproducibility of the work that we publish. This form provides structure for consistency and transparency in reporting. For further information on Nature Portfolio policies, see our Editorial Policies and the Editorial Policy Checklist.

Statistics

For all statistical analyses, confirm that the following items are present in the figure legend, table legend, main text, or Methods section.

n/a	Confirmed	
☐	☒	The exact sample size (*n*) for each experimental group/condition, given as a discrete number and unit of measurement
☐	☒	A statement on whether measurements were taken from distinct samples or whether the same sample was measured repeatedly
☐	☒	The statistical test(s) used AND whether they are one- or two-sided<br>*Only common tests should be described solely by name; describe more complex techniques in the Methods section.*
☒	☐	A description of all covariates tested
☒	☐	A description of any assumptions or corrections, such as tests of normality and adjustment for multiple comparisons
☐	☒	A full description of the statistical parameters including central tendency (e.g. means) or other basic estimates (e.g. regression coefficient) AND variation (e.g. standard deviation) or associated estimates of uncertainty (e.g. confidence intervals)
☐	☒	For null hypothesis testing, the test statistic (e.g. *F*, *t*, *r*) with confidence intervals, effect sizes, degrees of freedom and *P* value noted<br>*Give P values as exact values whenever suitable.*
☒	☐	For Bayesian analysis, information on the choice of priors and Markov chain Monte Carlo settings
☒	☐	For hierarchical and complex designs, identification of the appropriate level for tests and full reporting of outcomes
☒	☐	Estimates of effect sizes (e.g. Cohen's *d*, Pearson's *r*), indicating how they were calculated

Our web collection on statistics for biologists contains articles on many of the points above.

Software and code

Policy information about availability of computer code

Data collection	'R (R 4.0.2)' https//www.r-project.org/<br>'Fiji image analysis software (2.9.0)' (Schindelin et al., 2012) https://imagej.net/software/fiji/<br>'Zen2 (V2)' Zeiss https://www.zeiss.com/microscopy/us/products/microscope-software/zen.html<br>'Cell Ranger' (V2 & V3) (Zheng et al., 2017) https://support.10xgenomics.com/single-cell-gene-expression/software/pipelines/latest/what-is-cell-ranger
Data analysis	R (R 4.0.2) https://www.r-project.org/<br>Fiji image analysis software (2.9.0) (Schindelin et al., 2012) https://imagej.net/software/fiji/<br>Graphpad Prism (7.1.0 & 9.4.0) GraphPad https://www.graphpad.com/scientific-software/prism/<br>Illustrator Adobe (27.3.1) https://www.adobe.com/products/illustrator.html<br>Zen2 (V2) Zeiss https://www.zeiss.com/microscopy/us/products/microscope-software/zen.html<br><br>The following R packages were used for this study:<br>'Seurat (V3)' (Butler et al., 2018; Stuart et al., 2019)<br>'FGSEA (1.11.2)' (Korotkevich et al., 2021)<br>'Velocyto (0.6)' (La Manno et al., 2018)<br>'scVelo (0.2.4)' (Bergen et al., 2020)<br>'Bigscale2 (2.0)' (Iacono et al., 2018)

Additional software:
'Cytoscape_v3.8.1'
'BD FACS(TM) Software 1.2.0.142'

github web-link for custom codes:
'https://github.com/BeppuAN/20220912_GEO-asssession-GSE184384'

For manuscripts utilizing custom algorithms or software that are central to the research but not yet described in published literature, software must be made available to editors and reviewers. We strongly encourage code deposition in a community repository (e.g. GitHub). See the Nature Portfolio guidelines for submitting code & software for further information.

Data

Policy information about availability of data

All manuscripts must include a data availability statement. This statement should provide the following information, where applicable:
- Accession codes, unique identifiers, or web links for publicly available datasets
- A description of any restrictions on data availability
- For clinical datasets or third party data, please ensure that the statement adheres to our policy

The data discussed in this publication have been deposited in NCBI's Gene Expression Omnibus (Edgar et al., 2002) and are accessible through GEO Series accession number GSE184384 (https://www.ncbi.nlm.nih.gov/geo/query/acc.cgi?acc=GSE184384).

URL link to Github for code: https://github.com/BeppuAN/20220912_GEO-asssession-GSE184384

Human research participants

Policy information about studies involving human research participants and Sex and Gender in Research.

Reporting on sex and gender	N/A
Population characteristics	N/A
Recruitment	N/A
Ethics oversight	N/A

Note that full information on the approval of the study protocol must also be provided in the manuscript.

Field-specific reporting

Please select the one below that is the best fit for your research. If you are not sure, read the appropriate sections before making your selection.

[x] Life sciences [ ] Behavioural & social sciences [ ] Ecological, evolutionary & environmental sciences

For a reference copy of the document with all sections, see nature.com/documents/nr-reporting-summary-flat.pdf

Life sciences study design

All studies must disclose on these points even when the disclosure is negative.

Sample size	Sample size was determined based upon prior studies by us and others (Ray et al., 2016; Vaughan et al., 2015), and for this study are specified in figure legends. At least 3 biological replicates were used for all experiments with the exception of single cell RNAseq studies, the findings of which were validated using orthogonal approaches with sufficient biological replicates to determine significance.
Data exclusions	No data was excluded from the study.
Replication	With the exception of single cell RNAseq analysis, data from experiments presented in the manuscript have at least n = 3 biological replicates, and 1 independent experiment. Quantitative data derived from micrographs were repeated in an independent experiment at least once to confirm results prior to Quantification. Similar results were observed among all technical repeats that were performed.<br><br>For single cell RNAseq experiments, 5 biological replicates were pooled prior to cell sorting to account for technical variability associated with sample processing. We determined technical variability associated library preparation and sequencing to be negligible given that sample processing occurs on the same day and by the same technician. Single cell RNAseq experiments were not repeated due to high cost associated with the workflow.
Randomization	Animals were assigned to experimental groups based upon genotypes, with age distribution between postnatal 8-12 weeks and sex being randomly assigned covariates.

Blinding	Investigators were not blinded to sample allocation. Blinding was not feasible in experimental design due to personnel limitations.

Reporting for specific materials, systems and methods

We require information from authors about some types of materials, experimental systems and methods used in many studies. Here, indicate whether each material, system or method listed is relevant to your study. If you are not sure if a list item applies to your research, read the appropriate section before selecting a response.

Materials & experimental systems

n/a	Involved in the study
☐	☒ Antibodies
☐	☒ Eukaryotic cell lines
☒	☐ Palaeontology and archaeology
☐	☒ Animals and other organisms
☒	☐ Clinical data
☒	☐ Dual use research of concern

Methods

n/a	Involved in the study
☒	☐ ChIP-seq
☐	☒ Flow cytometry
☒	☐ MRI-based neuroimaging

Antibodies

Antibodies used

Primary antibodies for IF:
Chicken polyclonal anti-eGFP Abcam (Ab13970)
Chicken Polyclonal anti-Keratin 5 BioLegend (905901)
Mouse monoclonal anti-eGFP AF488 Santa Cruz (Sc-9996, clone: B-2)
Rat monoclonal anti-IL-22ra1 R&D systems (MAB42341, clone: 496514)
Goat polyclonal anti-tdTomato Scigen (Ab8181-200)
Goat polyclonal anti-p63 Santa Cruz (Sc-8609, clone: D-9)
Goat Polyclonal Ugrp1/Scgb3a2 R&D Systems (AF3465)
Syrian hamster Monoclonal anti-Pdpn LifeSpan Biosciences (LS-C143022-100, clone: 8.1.1)
Rabbit Polyclonal anti-Scgb1a1 Proteintech (10490-1-AP)
Rabbit Polyclonal anti-tdT Rockland (600-401-379)
Rabbit Polyclonal anti-Msln Thermo Fisher Scientific (PA5-79698)
Rabbit Polyclonal anti-Ltf Thermo Fisher Scientific (PA5-95513)
Rabbit Polyclonal anti-Bpifa1 Sigma-Aldrich (AV42475)
Rabbit polyclonal anti-Keratin 5 Cell Marque (EP1601Y)
Rabbit polyclonal anti-Keratin 5 Santa Cruz (Sc-66856, clone: H-40)
Rabbit polyclonal anti IL-22 Abcam (ab18499)
Rabbit polyclonal anti-Ki67 ebioscience (14-5698-82, clone: SolA15)

Secondary antibodies for IF:
Goat anti-Chicken Alexa Fluor 488 Thermo Fisher Scientific 6100-30
Goat anti-Hamster Alexa Fluor 488 Thermo Fisher Scientific A-21110
Donkey anti-Rabbit Alexa Fluor 488 Thermo Fisher Scientific A-21206
Donkey anti-Goat Alexa Fluor 555 Thermo Fisher Scientific A-21432
Donkey anti-Rabbit Alexa Fluor 555 Thermo Fisher Scientific A-31572
Goat anti-Chicken Alexa Fluor 568 Thermo Fisher Scientific A-11041
Donkey anti-Rat Alexa Fluor 594 Thermo Fisher Scientific A-21209
Donkey anti-Goat Alexa Fluor 594 Thermo Fisher Scientific A-11058
Donkey anti-Rabbit Alexa Fluor 594 Thermo Fisher Scientific A-21207
Goat anti-Hamster Alexa Fluor 594 Thermo Fisher Scientific A-21113
Goat anti-Chicken Alexa Fluor 647 Thermo Fisher Scientific A-21449
Donkey anti-Rabbit Alexa Fluor 647 Thermo Fisher Scientific A-31573
Donkey anti-Goat Alexa Fluor 647 Thermo Fisher Scientific A-21447

Primary antibodies for Flow:
Fitc rat monoclonal anti-CD45 BioLegend (103108, clone: 30-F11)
Fitc rat monoclonal anti-CD31 BioLegend (102406, clone: 390)
APC rat monoclonal anti-CD326 BioLegend (118218, clone: G8.8)
Pe/Cy7 rat monoclonal anti-CD326 BioLegend (118216, clone: G8.8)
Biotin monoclonal anti-CD31 BioLegend (102404, clone: 390)
Biotin monoclonal anti-CD45 BioLegend (103104, clone: 30-F11)
Biotin monoclonal anti-Ly-6A/E BioLegend (108112, clone: D7)

Primary antibodies for Cell Hashing

TotalSeq Hashtag 1 Antibody BioLegend (155801, clone: 30-F11)
TotalSeq Hashtag 2 Antibody BioLegend (155803, clone: 30-F11)
TotalSeq Hashtag 3 Antibody BioLegend (155805, clone: 30-F11)
TotalSeq Hashtag 4 Antibody BioLegend (155807, clone: 30-F11)

Validation

Antibodies used in this study have been validated by the corresponding manufacturer and in publications in which the antibodies were used. For FACS, positive staining was verified through use of compensation beads and fluorescence minus one (FMO) controls.

Primary antibodies for IF:
Chicken polyclonal anti-eGFP Abcam (Ab13970)
Product citations: 3182

Chicken Polyclonal anti-Keratin 5 BioLegend (905901)
Product citations: 2

Mouse monoclonal anti-eGFP AF488 Santa Cruz (Sc-9996, clone: B-2)
Product citations: 3182

Rat monoclonal anti-IL-22ra1 R&D systems (MAB42341, clone: 496514)
Product citations: 9

Goat polyclonal anti-tdTomato Scigen (Ab8181-200)
Product citations: 18

Goat polyclonal anti-p63 Santa Cruz (Sc-8609, clone: D-9)
Product citations: 42

Goat Polyclonal Ugrp1/Scgb3a2 R&D Systems (AF3465)
Product citations: 1

Syrian hamster Monoclonal anti-Pdpn LifeSpan Biosciences (LS-C143022-100, clone: 8.1.1)
Product citations: 1

Rabbit Polyclonal anti-Scgb1a1 Proteintech (10490-1-AP)
Product citations: 14

Rabbit Polyclonal anti-tdT Rockland (600-401-379)
Product citations: 3

Rabbit Polyclonal anti-Msln Thermo Fisher Scientific (PA5-79698)
Product citations: 1

Rabbit Polyclonal anti-Ltf Thermo Fisher Scientific (PA5-95513)
Antibody was validated by the manufacturer to be suitable for use in IHC.

Rabbit Polyclonal anti-Bpifa1 Sigma-Aldrich (AV42475)
Antibody was validated by the manufacturer to be suitable for use in IHC.

Rabbit polyclonal anti-Keratin 5 Cell Marque (EP1601Y)
Product citations: 7

Rabbit polyclonal anti-Keratin 5 Santa Cruz (Sc-66856, clone: H-40)
Product citations: 4

Rabbit polyclonal anti IL-22 Abcam (ab18499)
Antibody was validated by the manufacturer to be suitable for use in IHC.

Rabbit polyclonal anti-Ki67 ebioscience (14-5698-82, clone: SolA15)
Product citations:306

Primary antibodies for Flow:
Fitc rat monoclonal anti-CD45 BioLegend (103108, clone: 30-F11)
Product citations: 235

Fitc rat monoclonal anti-CD31 BioLegend (102406, clone: 390)
Product citations: 38

APC rat monoclonal anti-CD326 BioLegend (118218, clone: G8.8)

Product citations: 77

Pe/Cy7 rat monoclonal anti-CD326 BioLegend (118216, clone: G8.8)
Product citations: 77

Biotin monoclonal anti-CD31 BioLegend (102404, clone: 390)
Product citations: 29

Biotin monoclonal anti-CD45 BioLegend (103104, clone: 30-F11)
Product citations: 52

Biotin monoclonal anti-Ly-6A/E BioLegend (108112, clone: D7)
Product citations: 51

Primary antibodies for Cell Hashing:
TotalSeq Hashtag 1 Antibody BioLegend (155801, clone: 30-F11)
Product citations: 13

TotalSeq Hashtag 2 Antibody BioLegend (155803, clone: 30-F11)
Product citations: 11

TotalSeq Hashtag 3 Antibody BioLegend (155805, clone: 30-F11)
Product citations: 9

TotalSeq Hashtag 4 Antibody BioLegend (155807, clone: 30-F11)
Product citations: 8

Eukaryotic cell lines

Policy information about cell lines and Sex and Gender in Research

Cell line source(s)	MLg cells were obtained from ATCC, expanded under recommended culture conditions, and co-cultured with primary mouse lung epithelial cells to establish epithelial organoid cultures.
Authentication	No cell line authentication was performed.
Mycoplasma contamination	Cultured cells were not tested for mycoplasma contamination.
Commonly misidentified lines (See ICLAC register)	No commonly misidentified lines were used in this study.

Animals and other research organisms

Policy information about studies involving animals; ARRIVE guidelines recommended for reporting animal research, and Sex and Gender in Research

Laboratory animals	Sftpc-CreER, Source: Jackson Labs; (Rock et al., 2011), strain ID: 028054, age:8-12<br>Scgb1a1-CreER, Source: Jackson Labs; (Rawlins et al., 2009), strain ID: 016225, age:8-12<br>Krt5-CreER, Source: Jackson Labs; (Van Keymeulen et al., 2011), strain ID: 029155, age:8-12<br>IL-22Cre, Source: Jackson Labs; (Ahlfors et al., 2014), strain ID:027524, age:8-12<br>IL-22ra1fl/fl, Source: Jackson Labs; (Zheng et al., 2016), strain ID:031003, age:8-12<br>Shh-Cre, Source: Jackson Labs; (Harfe et al., 2004), strain ID:005622, age:8-12<br>ROSA-26-mTmG, Source: Jackson Labs; (Muzumdar et al., 2007), strain ID:007576, age:8-12<br>ROSA-26-tdT, Source: collaborator; (Madisen et al., 2010), strain ID:007905, age:8-12<br>RC::RLTG, Source: Jackson Labs;(Plummer et al., 2015), strain ID:026931, age:8-12<br>Scgb3a2-DreER, Source: Jackson Labs NA – novel line, age:8-12<br><br>Housing for mice complied with IUCAC guidelines.<br>Light/dark cycle: 12:12h<br>Temperature: 68-79 degrees F<br>Humidity: 30-70%
Wild animals	This study did not involve wild animals.
Reporting on sex	A mixture of male and female mice were used for each experiment.
Field-collected samples	Study did not include samples collected from the field.

Ethics oversight

All studies using mice were performed using protocols that were approved by the Cedars-Sinai Institutional Animal Care and Use Committee and by the Institutional Biosafety Committee.

Note that full information on the approval of the study protocol must also be provided in the manuscript.

Flow Cytometry

Plots

Confirm that:

- [x] The axis labels state the marker and fluorochrome used (e.g. CD4-FITC).
- [x] The axis scales are clearly visible. Include numbers along axes only for bottom left plot of group (a 'group' is an analysis of identical markers).
- [x] All plots are contour plots with outliers or pseudocolor plots.
- [x] A numerical value for number of cells or percentage (with statistics) is provided.

Methodology

Sample preparation

Preparation of single cell suspensions for Single cell RNAseq:

Type II & immune subsets: Mouse lung biopsies were collected at the indicated time points: naïve, 3, 5, 7, 9, 11, 14, 17, 21, 60, 120 and 240 days post infection. A sample size of 5 C57/Bl6 WT mice were used for ¬each timepoint. Cell suspensions from each condition were pooled together prior to cell sorting. On the day of biopsy collection, the entire mouse lung was separated from the chest cavity and stored in a conical containing 4o C 1XHBSS. Isolated lung lobes were intratracheally instilled with a 3ml mixture containing 1U elastase/1ml 1XHBSS for 30 minutes at 37o C. The crude cell suspension underwent mechanical agitation prior to incubation in dissociation solution with a final composition of 1X Liberase/1X HBSS for 30 minutes at 37oC. Dissociation buffer was quenched with a solution containing 2% FBS/1mM EDTA/1X HBSS on ice. Cells were filtered through a 70µm nylon mesh to remove undigested tissue. Cells were centrifuged at 500xg for 10 minutes and resuspended in 1ml blood cell lysis solution for 1 minute to remove red blood cells from the suspension. Red blood cell lysis buffer was quenched using 25 ml 2% FBS/1mM EDTA/1X HBSS, followed by centrifugation at 500 x g for 10 minutes to pellet intact cells. When isolating epithelium, cells were resuspended in 1ml 2% FBS/1mM EDTA/1X HBSS and magnetic bed separation was performed to deplete CD31 and CD45 subsets.

Conducting airway subsets: Single-cell RNA seq data was generated from cell suspensions enriched for conducting airway epithelium. The aforementioned cell suspension prep was performed on TM inoculated Sftpc-CreER/ROSA-mTmG and a gating strategy was used to enrich for tdT negative epithelium (i.e. CD326+CD45-CD31-tdT-) by FACS. For these experiments, a sample size of 3 Sftpc-CreER/ROSA-mTmG were pooled together prior to cell sorting. Cells were collected at the same indicated time points (naïve, 3, 5, 7, 9, 11, 14, 17, 21, 60, 120 and 240 days post infection) and a minimum of 50,000 cells were collected per timepoint. In experiments where isolation of conducting airway epithelium was not possible using Sftpc-CreER/ROSA-mTmG (Fig. 7H) an alternative gating strategy was used to deplete for Type II cells. A combination of Sca-1 and CD24 antibodies were used in lieu of the tdT lineage tag (i.e CD326+CD45-CD31-Sca1+CD24+).

Instrument

BD influx cell sorter

Software

BD FACS(TM) Sortware 1.2.0.142

Cell population abundance

Type II & immune subsets: A minimum of 50,000 epithelial cells and 100,000 immune cells were sorted for each timepoint.

Conducting airway subsets: A minimum of 50,000 epithelial cells were sorted for each timepoint.

Gating strategy

Type II & immune subsets: Cell suspensions were enriched for epithelial and immune populations by FACS, with selection for CD326+CD45-CD31- and CD326-CD45+CD31- to yield enriched epithelial and immune cell fractions, respectively.

Conducting airway subsets: Cell suspensions were enriched for epithelial populations by FACS, with selection for CD326 +CD45-CD31- to yield enriched epithelial fractions.

- [x] Tick this box to confirm that a figure exemplifying the gating strategy is provided in the Supplementary Information.
